# Bayesian Hierarchical Random Effects Models in Forensic Science

**DOI:** 10.3389/fgene.2018.00126

**Published:** 2018-04-16

**Authors:** Colin G. G. Aitken

**Affiliations:** School of Mathematics and Maxwell Institute, The University of Edinburgh, Edinburgh, United Kingdom

**Keywords:** Bayes' Theorem, evidence evaluation, forensic science, hierarchical models, likelihood ratios, random effects, SAILR, statistics

## Abstract

Statistical modeling of the evaluation of evidence with the use of the likelihood ratio has a long history. It dates from the Dreyfus case at the end of the nineteenth century through the work at Bletchley Park in the Second World War to the present day. The development received a significant boost in 1977 with a seminal work by Dennis Lindley which introduced a Bayesian hierarchical random effects model for the evaluation of evidence with an example of refractive index measurements on fragments of glass. Many models have been developed since then. The methods have now been sufficiently well-developed and have become so widespread that it is timely to try and provide a software package to assist in their implementation. With that in mind, a project (SAILR: Software for the Analysis and Implementation of Likelihood Ratios) was funded by the European Network of Forensic Science Institutes through their Monopoly programme to develop a software package for use by forensic scientists world-wide that would assist in the statistical analysis and implementation of the approach based on likelihood ratios. It is the purpose of this document to provide a short review of a small part of this history. The review also provides a background, or landscape, for the development of some of the models within the SAILR package and references to SAILR as made as appropriate.

## 1. Introduction

Statistical analyses for the evaluation of evidence have a considerable history. It is the purpose of this document to provide a short review of a small part of this history. It brings together ideas from the last forty years for statistical models when the evidence is in the form of measurements and thus of continuous data. The data are also hierarchical with two levels. The first level is that of source, the origin of the data. The second level is of items within a source. The models used to represent the variability in the data are random effects models. The models are chosen from analyses of samples of sources from some relevant population. Finally, the analysis is Bayesian in nature with prior distributions for the parameters of the within-source distributions. The nature of the prior distributions is informed from training data based on the samples from the relevant population.

The remainder of the document is structured as follows. Section 2 provides a general introduction to the likelihood ratio as a measure of the value of evidence. Section 3 provides a framework for models for comparison and discrimination. Section 4 discusses the assessment of model performance. An Appendix gives formulae for some of the more commonly used models.

## 2. The value of evidence

Part of the role of a forensic scientist is to interpret evidence found at a crime scene in order to aid fact-finders in a criminal case (e.g., the judge or jury) in their decision making. The forensic scientist may be asked to comment on the value of the evidence in the context of various competing statements about the evidence, each of which may be true or false.Generally, a forensic scientist must consider two competing statements relating to the evidence, one put forward by the prosecution in a criminal case, and one put forward by the defense (Cook et al., 1998b). These statements are known as *propositions*^1^. They generally come in pairs that are mutually exclusive, though not necessarily exhaustive. For a debate about the requirement, or otherwise, for the propositions to be exhaustive (see Biedermann et al., 2014; Fenton et al., 2014a,b).

One member of the pair is associated with the prosecution and conventionally denoted *H*_*p*_. The other member of the pair is associated with the defense and conventionally denoted *H*_*d*_. The evidence to be evaluated is denoted *E*^2^. The value of evidence is taken to be the relative values of the probability of the evidence if a proposition put forward by the prosecution is true and the probability of the evidence if a proposition put forward by the defense is true. However, evidence is not evaluated in isolation. There is always other information to be taken into account, including, for example, personal knowledge of the fact-finder. Denote this information by *I*. The value of the evidence, denoted *V* say, can then be written formulaically as

$$V=\frac{\mathrm{Pr}(E|H_{p},I)}{\mathrm{Pr}(E|H_{d},I)},$$

where Pr denotes *Probability*. This ratio is known as the *likelihood ratio*.

The likelihood ratio is the method used by SAILR to evaluate evidence. SAILR (Software for the Analysis and Implementation of Likelihood Ratios) is a user-friendly Graphical Interface (GUI) to calculate numerical likelihood ratios in forensic statistics and its development under the direction of the Netherlands Forensic Institute (NFI) was funded by the European Network of Forensic Science Institutes through their Monopoly programme. The likelihood ratio is a generally accepted measure for the value of evidence in much forensic case-work.

This representation of the value of evidence has a very good intuitive interpretation. Consider the odds form of Bayes' Theorem in the forensic context of the evaluation of evidence. The odds form of Bayes' Theorem then enables the prior odds(i.e., prior to the presentation of *E*) in favor of the prosecution proposition *H*_*p*_ relative to the defense proposition *H*_*d*_ to be updated to posterior odds given *E*, the evidence under consideration. This is done by multiplying the prior odds by the likelihood ratio. The odds form of Bayes' Theorem may then be written as

(1) $$\frac{\mathrm{Pr}(H_{p}|E,I)}{\mathrm{Pr}(H_{d}|E,I)}=\frac{\mathrm{Pr}(E|H_{p},I)}{\mathrm{Pr}(E|H_{d},I)}\times \frac{\mathrm{Pr}(H_{p}|I)}{\mathrm{Pr}(H_{d}|I)}.$$

The likelihood ratio (LR) is the ratio

(2) $$\frac{\mathrm{Pr}(H_{p}|E,I)/\mathrm{Pr}(H_{d}|E,I)}{\mathrm{Pr}(H_{p}|I)/\mathrm{Pr}(H_{d}|I)}$$

of posterior odds to prior odds. It is the factor which converts the prior odds in favor of the prosecution proposition to the posterior odds in favor of the prosecution proposition. The representation in Equation (1) also emphasizes the dependence of the prior odds on other information *I*. Values of the *LR* > 1 are supportive of *H*_*p*_, the proposition put forward by the prosecution. Values of the *LR* < 1 are supportive of *H*_*d*_, the proposition put forward by the defense. The word “odds” should be used advisedly. If *H*_*p*_ and *H*_*d*_ are not exhaustive then the component probabilities Pr(*H*_*p*_ ∣ *E, I*) and Pr(*H*_*d*_ ∣ *E, I*) cannot be derived from this ratio. All that can be said is that the posterior ratio is different from the prior ratio by a factor *V*.

An advantage of this formulation of evidence evaluation is the ease with which the effect of the addition of new evidence can be determined. The posterior odds for one piece of evidence, *E*_1_ say, can be the prior odds for a second piece of evidence, *E*_2_ say. Then Equation (1) may be rewritten as

(3) $$\frac{\mathrm{Pr}(H_{p}|E_{1},E_{2},I)}{\mathrm{Pr}(H_{d}|E_{1},E_{2},I)}=\frac{\mathrm{Pr}(E_{2}|H_{p},E_{1},I)}{\mathrm{Pr}(E_{2}|H_{d},E_{1},I)}\times \frac{\mathrm{Pr}(H_{p}|E_{1},I)}{\mathrm{Pr}(H_{d}|E_{1},I)},$$

where the conditioning of the evaluation of *E*_2_ on *E*_1_ is made explicit.

An illustration of the effect of evidence with a value V of 1,000 on the odds in favor of *H*_*p*_ relative to *H*_*d*_ is given in Table 1.

**Table 1:** Effect on prior odds in favor of *H*_*p*_ relative to *H*_*d*_ of evidence *E* with value *V* of 1,000.

**Prior odds *Pr*(*H*_*p*_)/*Pr*(*H*_*d*_)**	**V**	**Posterior odds *Pr*(*H*_*p*_∣*E*)/*Pr*(*H*_*d*_∣*E*)**
1/10,000	1,000	1/10
1/100	1,000	10
1 (evens)	1,000	1,000
100	1,000	100,000

*Reference to background information I is omitted*.

The following quote is very pertinent.

‘That approach does not ask the jurors to produce any number, let alone one that can qualify as a probability. It merely shows them how a “true” prior probability would be altered, if one were in fact available. It thus supplies the jurors with as precise and accurate an illustration of the probative force of the quantitative data as the mathematical theory of probability can provide. Such a chart, it can be maintained, should have pedagogical value for the juror who evaluates the entire package of evidence solely by intuitive methods, and who does not himself attempt to assign a probability to the “soft” evidence.’ Kaye (1979).

The “it” in this context is a chart depicting, in numerical terms, how much the prior odds in favor of a proposition is enhanced by the evidence being evaluated. This is a graphical equivalent of Table 1. The mathematical tool for devising such a chart is Bayes' Theorem. These remarks of Kaye's refer to characteristics of the general method for the evaluation of evidence that is the likelihood ratio. They do not refer to a particular case. For example, it is not possible to comment on the accuracy of a likelihood ratio estimation in a particular case because the true value of the likelihood ratio is not known nor can it be known. It is, however, possible to refer to the accuracy of a method and performance assessment in general is discussed in section 4.

The use of a likelihood ratio for the evaluation of evidence is not a new idea. In the Dreyfus case (Champod et al., 1999), it was argued that

… since it is absolutely impossible for us [the experts] to know the *a priori* probability, we cannot say: this coincidence proves that the ratio of the forgery's probability to the inverse probability is a real value. We can only say: following the observation of this coincidence, this ratio becomes *X* times greater than before the observation (Darboux et al., 1908).

The “ratio” in this quotation is the odds in favor of one proposition over another, The *X* refers to the likelihood ratio. The posterior odds in favor of the proposition is then *X* times the prior odds.

The ideas were also used in the work of I.J. Good and A.M. Turing as code-breakers at Bletchley Park during World War II (Good, 1979).

### 2.1. Background information

The likelihood ratio updates the prior odds, those before consideration of evidence *E*, to posterior odds, which take *E* into account. The posterior odds are the odds with which, ultimately, the fact-finder is concerned. If the likelihood ratio multiplied by the prior odds is larger than one, then the probability of *H*_*p*_ given the evidence is larger than that of *H*_*d*_ given the evidence. As these propositions may not be exhaustive their explicit values, rather than their relative value, may not be known. It is the responsibility of the fact-finder to determine a value for the prior odds. The prior odds can then be combined with the likelihood ratio to obtain posterior odds. A forensic scientist is concerned only with the value of the evidence as expressed by the likelihood ratio so cannot usually comment on the value of the posterior odds. The likelihood ratio is considered as the strength of support of the evidence for one of the two propositions *H*_*p*_ or *H*_*d*_.

The application of this form to a specific case is crucially dependent on the background information *I*. However, the background information available to each person is different. In part, this is because each person is different. In part it is because of professional differences. The information that a forensic scientist should use for their determination of the likelihood ratio is different from that which a fact-finder, such as judge or jury member, should use for their determination of the odds in favor of the prosecution proposition. There are differences in the background information available to these participants in the judicial process but these differences have no effect on the posterior odds in favor of the prosecution proposition.

Let *I* = *I*_*a*_ ∪ *I*_*b*_ where *I*_*a*_ is background information available to the forensic scientist and *I*_*b*_ is background information available to the fact-finder. There will be information available to both, the intersection *I*_*a*_ ∩ *I*_*b*_ is not empty. It can then be shown (Aitken and Nordgaard, 2017) that the posterior odds may be written in the form

$$\frac{\mathrm{Pr}(H_{p}|E,I)}{\mathrm{Pr}(H_{d}|E,I)}=\frac{\mathrm{Pr}(E|H_{p},I_{b})}{\mathrm{Pr}(E|H_{d},I_{b})}\times \frac{\mathrm{Pr}(H_{p}|I_{a})}{\mathrm{Pr}(H_{d}|I_{a})}.$$

The fact-finder and the forensic scientist have to treat the common information (*I*_*a*_ ∩ *I*_*b*_) with appropriate discretion.

### 2.2. Uniqueness of the likelihood ratio

The role of the likelihood ratio as the factor that updates the prior odds to the posterior odds has a very intuitive interpretation. There is also a mathematical derivation that shows it, or a function of it such as the logarithm, is the only way to update evidence. It was shown many years ago by I.J.Good in two brief notes in the *Journal of Statistical Computation and Simulation* (Good, 1989a,b) repeated in Good (1991) and in Aitken and Taroni (2004) that, with some very reasonable assumptions, the assessment of uncertainty inherent in the evaluation of evidence leads inevitably to the likelihood ratio as the only way in which this can be done.

Consider evidence *E* which it is desired to evaluate in the context of two mutually exclusive propositions *H*_*p*_ and *H*_*d*_. Denote the value of the evidence by *V*. As always, the value will depend on background information *I* but this will not be stated explicitly. There are other assumptions implicit in this approach, namely that there is a probability that can be associated with evidence and one that is dependent on propositions and only on propositions (and background information). Another assumption is that *V* is a function only of the probability of *E*, given *H*_*p*_ to be true, and of the probability of *E*, given *H*_*d*_ to be true.

Let *x* = Pr(*E* ∣ *H*_*p*_) and *y* = Pr(*E* ∣ *H*_*d*_) where *I* is omitted for ease of notation. The assumption that *V* is a function only of these probabilities can be represented mathematically as

V=f(x,y)

for some function *f*.

Now, consider another piece of evidence *T* which is irrelevant to *E*, to *H*_*p*_ and to *H*_*d*_. Irrelevance is taken in the probabilistic context to be equivalent to independence so that *T* may be taken to be independent of *E*, of *H*_*p*_ and of *H*_*d*_. It is then permissible for Pr(*T*) to be given notation which does not refer to any of *E, H*_*p*_ or *H*_*d*_. Thus, let Pr(*T*) be denoted by θ. Then

$$\begin{array}{l} \mathrm{Pr}(E,T|H_{p})=\mathrm{Pr}(E|H_{p})\mathrm{Pr}(T|H_{p})\text{ by the independence of }E\text{ and }T\\ \;=\mathrm{Pr}(E|H_{p})\mathrm{Pr}(T)\text{ by the independence of }T\text{and }H_{p}\\ \;=x\theta .\; \end{array}$$

Similarly,

$$\mathrm{Pr}(E,T|H_{d})=y\;\theta .$$

The value of (*E, T*) is *f*(θ*x*, θ*y*) by the definition of *f*. However, evidence *T* is irrelevant and has no effect on the value of evidence *E*. Thus, the value of the combined evidence (*E, T*), *f*(θ*x*, θ*y*), is equal to the value *V* of *E, f*(*x, y*), and

V=f(x,y)=f(θx,θy)

for all θ in the interval [0,1] of possible values of Pr(*T*).

The only class of functions of (*x, y*) for which this can be said to be the case is the class which are functions of *x*/*y* or

$$\mathrm{Pr}(E|H_{p})/\mathrm{Pr}(E|H_{d})$$

which is the likelihood ratio. Hence the value *V* of evidence has to be a function of the likelihood ratio. It has been argued (Lund and Iyer, 2017) that the forensic community view the likelihood ratio as only one possible tool for communication with decision makers. The argument of Good shows that it is the only logically admissible form of evaluation.

### 2.3. Weight of evidence

An interesting note of terminology can be mentioned here. It is common in some legal circles to talk of the *weight of evidence*. The concept of weight of evidence is an old idea. The term *weight of evidence* should be used for the logarithm of the likelihood ratio. The terminology was first used by Peirce (1878). The likelihood ratio is the *value* of the evidence and its logarithm is the *weight* of the evidence. The logarithm of the likelihood ratio has the pleasingly intuitive operation of additivity when converting the logarithm of the prior odds in favor of a proposition to the logarithm of the posterior odds in favor of the proposition.

(4) $$\log\left\{\frac{\mathrm{Pr}(H_{p}|E)}{\mathrm{Pr}(H_{d}|E)}\right\}=\log\left\{\frac{\mathrm{Pr}(E|H_{p})}{\mathrm{Pr}(E|H_{d})}\right\}+\log\left\{\frac{\mathrm{Pr}(H_{p})}{\mathrm{Pr}(H_{d})}\right\},$$

with *I* omitted. When considering the scales of justice it is the logarithm of the probabilities of the evidence given each of the two competing propositions that should be put in the scales, not the probabilities.

### 2.4. Terminology for evidence

The evidence under consideration in this document and within the SAILR project is evidence that could have been transferred either from the crime scene to the criminal or from the criminal to the crime scene. Evidence that could have been so transferred is in the form of traces. Thus it has two names *transfer* or *trace* evidence. The evidential material discussed here is in the form of individual items. Thus, there may be a finite number of items, such as tablets or sachets of drugs or fragments of glass. Alternatively, the evidence may be a single measurement such as that of a DNA profile.

Consider the situation in which a crime has been committed, there is a crime scene and the investigation has reached the stage where a suspect has been identified. Trace evidence, denoted *E*, of a particular type has been found at the crime scene and on the suspect and its value is of interest. The evidence *E* may be partitioned into two parts, that found at the crime scene and that found in association with the suspect. In practice, the terminology takes a different form which depends on whether the source of the evidence is known or not known. A distinction is also drawn between evidential material and the evidence for evaluation. Evidence for evaluation is the observations made on the material. Only evidence which is in the form of measurements and thus represented by continuous data is considered here. Other factors such as the locations in which the material was found and the quantity of the material are not considered. Evidence of a discrete nature such as binary data as in the presence or absence of striation marks is also not considered.

Evidence whose source is known is called *control* evidence *E*_*c*_. Evidence whose source is not known is called *recovered* evidence *E*_*r*_. Measurements on *E*_*c*_ are conventionally denoted **x** where **x** = (*x*_1_, …, *x*_*m*_) are *m* sets of measurements and where *x*_*i*_, *i* = 1, …, *m* may be univariate or multivariate. Measurements on *E*_*r*_ are conventionally denoted **y** where **y** = (*y*_1_, …, *y*_*n*_) are *n* sets of measurements and where *y*_*j*_, *j* = 1, …, *n* may be univariate or multivariate ^3^.

For an evaluative comparison of **x** and **y**, background data **z** are needed. These background data should be a representative sample of all possible sources from the population of interest, known as the *relevant* population. Ideally, the sample should be a random sample but this is rarely possible for practical reasons. The sample is often what might be called a *convenience* sample. If the convenience sample can be demonstrated to be composed of sources chosen in a manner independent of the case under investigation then the inference based on the comparison of **x** and **y** informed on **z** should be valid. Computation of the likelihood ratio requires data files from **x**, **y** and **z**.

One example of evidence in the form of multivariate data relates to glass elemental content. Such data are often subjected to a logarithmic transformation after taking the ratios of a particular elemental content to the oxygen content, for example, log_10_(*NaO*) = log_10_(*Na*/*O*). These measurements can be for each of *m* fragments of control evidence and for each of *n* fragments of recovered evidence (Zadora et al., 2014). This evidence can be multivariate as there can be several ratios measured for each fragment, e.g., log_10_(*NaO*), log_10_(*MgO*) and log_10_(*AlO*). The control evidence is the measurements from a number *m* of fragments of glass from a broken window at a crime scene; the source of the fragments is known to be the window, items within source are the fragments. The recovered evidence is the measurements from a number *n* of fragments of glass found in association with a suspect, for example on clothing identified as theirs. The source of the fragments of glass from the suspect is unknown. It may or may not have come from the window at the crime scene. A second example could be the measurements of color chromaticity coordinates on fibers and the evidence is bivariate (Martyna et al., 2013). There are three color chromaticity coordinates. The sum of their values is fixed so given the values of any two, the third is known. Control evidence is the measurements of color chromaticity coordinates from a number *m* of fibers from an article of clothing belonging to a suspect; the source is the article, the items are the fibers. Recovered evidence is the measurements of color chromaticity coordinates from a number *n* of fibers found at a crime scene. Thus control evidence may be found at a crime scene or in association with a suspect. Similarly, recovered evidence may be found at a crime scene or in association with a suspect.

Often the number *m* of control items can be chosen by the investigator. The number *n* of recovered items may be determined by what is available and the investigator has little choice in the selection of this number. If the number of recovered items is large, in some sense, and perhaps so large as for it to be impractical to count or analyse them, then the investigator may decide to select *n* items where *n* is less than the number available. Procedures for the choice of *n* and the manner of selection of the items are not discussed in this document or SAILR other than to note that the evidence selected should be representative of the total evidence available as far as is possible. Further information is available in Aitken and Taroni (2004) and references therein.

The likelihood ratio V for the comparison of {**x, y**} where *E* is replaced by {**x, y**} is then

(5) $$\text{V}=\frac{\mathrm{Pr}(x,y|H_{p})}{\mathrm{Pr}(x,y|H_{d})},$$

where again the conditioning on *I*, the background information, has been omitted for clarity of notation.

Often, the propositions being considered are *H*_*p*_ that the control and recovered evidence are from the same source and *H*_*d*_ that the control and recovered evidence are from different sources. In such a circumstance, **x** and **y** may be assumed independent if *H*_*d*_ is true as they come from different sources. Then Equation (5) may be written as

(6) $$\text{V}=\frac{\mathrm{Pr}(x,y|H_{p})}{\mathrm{Pr}(x|H_{d})\mathrm{Pr}(y|H_{d})}.$$

If **x** and **y** are continuous data, as is the case when the evidence is in the form of measurements rather than counts, the probabilities in the numerator and denominator are replaced by probability density functions, denoted say *f*(**x, y**) for the joint density and *f*(**x**) and *f*(**y**) for the marginal distributions. The continuous analog of Equation (6) can then be written as

(7) $$\text{V}=\frac{f(x,y|H_{p})}{f(x|H_{d})f(y|H_{d})}.$$

In most cases, the full specification of the probability density function is unknown. The form of the distribution may be known or a reasonable assumption of its form may be made. For example, it may be known or can be assumed that the appropriate distribution is a Normal distribution. This assumption may be based on the unimodal, symmetric nature of the distribution. If the distribution has a positive skew then a transformation to normality with a logarithmic transformation of the data may be possible before consideration of the likelihood ratio. However, the parameters may neither be known nor able to be assumed known.

The numerator of Equation (7) may be written as *f*(**x, y** ∣ *H*_*p*_) = *f*(**y** ∣ **x**)∣*H*_*p*_)*f*(**x** ∣ *H*_*p*_). Since the distribution of **x** is independent of whether *H*_*p*_ or *H*_*d*_ is true, *f*(**x** ∣ *H*_*p*_) = *f*(**x** ∣ *H*_*d*_) and Equation (5) may be written as

$$f(y|x,H_{p})/f(y|H_{d}).$$

See Equation (18) in Appendix for an example.

### 2.5. Training data

When parameters are not known, information about their possible values may be obtained from data independent of the crime but thought to be relevant for consideration of the variability in the measurements of the data comprising the evidence. These data are the *training data* or *background data* and are conventionally denoted **z**. These data are considered to be a sample from a population, known as a *relevant* population. There is considerable continuing debate as to how to choose a population that is relevant for a particular crime and, once chosen, how a sample may be chosen from it to be a representative sample of the population. See, for example, *R. v. T* [2010] EWCA 2439, where the debate related to the choice of populations of shoes relevant for the consideration of evidence of shoeprints. Often the sample is a convenience sample; see section 2.4.

An alternative procedure would be to sample anew each time from a population deemed relevant to the case under investigation. A relatively early example of this is the investigation of a murder in Biggar, a town near Edinburgh, in 1967. A bite mark found on the breast of a young girl who had been murdered had certain characteristic marks, indicative of the conformation of the teeth of the person who had bitten her. A 17-year-old boy was found with this conformation and he became a suspect. Examination of 90 other boys of the suspect's age showed that the particular conformation was not at all common. The 90 other boys could be considered as a sample from a relevant population. Further details are available in Harvey et al. (1968). However, in most individual investigations it is not practical to obtain such a bespoke relevant population.

### 2.6. Hierarchy of evidence

Often, with measurements, the training data can be thought of as a set of sources of items. Measurements are made of one or more characteristics of the items. For example, consider again the composition of the elemental ratio of various elements of glass to oxygen for glass fragments from a set of windows. The items are glass fragments. A source would be a window. The training set is a set of windows. The set of windows is a sample from some population of windows, deemed relevant for crimes involving windows. The measurements are said to be *hierarchical* with two levels. One level is the fragment of glass within a window. Variation amongst measurements of fragments within a window is known as *within-group* or *within-source variation*. The second level is the window. Variation amongst measurements between windows is known as between-group or between-source variation. Measurements are taken from an item (fragments of glass) within a source (window). Notationally, the training data **z** has two indices, one for each level and may be represented as **z** = {*z*_*kℓ*_; *k* = 1, …, *g*, ℓ = 1, …, *h*} where *g* is the number of sources in the training set and *h* is the number of measurements within sources. The number of measurements within sources need not necessarily be constant though it is computationally convenient if this can be arranged during the compilation of the training set. Occasionally there may be further levels, for example measurement error.

### 2.7. Propositions

As well as evidence (*E*) and background information *I*, evidence evaluation depends on propositions *H*_*p*_ and *H*_*d*_. There are different types of propositions, also known as *levels*. Both propositions (*H*_*p*_ and *H*_*d*_) in any particular situation for the evaluation of evidence are at the same level. There are four different levels of propositions, known respectively as *offense* level, *activity* level, *source* level and *sub-source* level (Cook et al., 1998a; Evett et al., 2000).

The levels, with examples, are described as follows.

- *Offense level*: the propositions may be that the defendant is guilty of an offense (truly guilty, not just declared guilty) and that the defendant is innocent (truly innocent, not just declared not guilty).
- *Activity level*: the propositions concern an activity by the defendant which may or may not be a criminal act. An example of a pair of activity level propositions could be that the defendant hit the victim and that the defendant did not hit the victim.
- *Source level*: the propositions concern the source of evidential material. There is no consideration of the activity that may have led to the material being where it was found. An example of a pair of source level propositions could be that blood found at the scene of a crime came from the defendant and that the blood found at the scene of the crime came from some other source, unrelated to the defendant. Note that this example is one in which the two propositions are not exhaustive; relatives of the defendant are not included. SAILR can only be used for likelihood ratio computation on source level.
- *Sub-source level*: the propositions concern material for which it is not possible to identify a source. An example of a pair of sub-source level propositions could be that DNA found at a crime scene came from the defendant and that DNA found at the crime scene came from some other source, unrelated to the defendant. The quantity of material found is insufficient to identify its source, e.g., whether it came from blood or semen.

## 3. Framework for models

The likelihood ratio may be used in the context of forensic science in two different ways, that of comparison and that of discrimination. For comparison, two pieces of evidence found in different places are compared to see if they had a common source. For discrimination, one piece of evidence is compared with several sets of training or background data from different sources to see from which source the evidence may have come.

Most of the models described here are so-called *feature-based models*. These are models developed from the measurements (features) on the evidential material. Other models described are so-called *score-based models*. There may be occasions with multivariate data when a feature-based model is not tractable, e.g., multidimensional binary data where the number of possible models is unmanageable. On such occasions, the distance, denoted *d*(**x**, **y**), between control (**x**) and recovered (**y**) data can be used instead.

### 3.1. Comparison for feature-based models

#### 3.1.1. The likelihood ratio approach for continuous univariate evidential data with normal distributions for the means and known variances

A common problem occurs in forensic science when the prosecution and defense propositions concern whether two objects are from the same source or from different sources. For example, if a glass fragment is found on a suspect and there is a broken window at the crime scene, one proposition might be that the glass fragment found on the suspect came from the window at the crime scene, and the other proposition might be that the glass fragment came from some other window. The evidence is given by a set of measurements from the glass fragment found on the suspect (the recovered sample) and a set of measurements from one or more glass fragments from the crime scene (the control sample). The problem is one of comparison.

The structure of these models reflects the hierarchical nature of the underlying data (measurements and variation within a source and then variation between sources). Using a distribution for the means θ_1_ and θ_2_ in this way accounts for variance within source (σ^2^) and variance between sources (τ^2^).

The problem for the fact-finder is to determine which of the two propositions (*H*_*p*_ or *H*_*d*_) is more likely, given all of the evidence in the case. Denote the other evidence and background information by *I* as before. The fact-finder can consider which proposition is more likely by considering the relative size of the two probabilities $\mathrm{Pr}\left(H_{p}\;|\;\bar{x},ȳ,I\right)$ and $\mathrm{Pr}\left(H_{d}\;|\;\bar{x},ȳ,I\right)$ (technically, in cases where the statistical assumptions include knowledge of the variances σ^2^ and τ^2^ and of a Normal distribution for the measurements, the means of the control and recovered samples are sufficient statistics so can be used in place of the measurements **x** and **y**). Let $f\left(\bar{x},ȳ\;|\;H_{p},I\right)$ be the joint probability density function of $\bar{x}$ and ȳ, given proposition *H*_*p*_ and *I* and let $f\left(\bar{x},ȳ|H_{d},I\right)$ be the joint probability density function of $\bar{x}$ and ȳ given proposition *H*_*d*_ and *I*. In this context Equation (1) may be represented as

(8) $$\frac{P(H_{p}|\bar{x},\bar{y},I)}{P(H_{d}|\bar{x},\bar{y},I)}=\frac{f(\bar{x},\bar{y}|H_{p},I)}{f(\bar{x},\bar{y}|H_{d},I)}\times \frac{P(H_{p}|I)}{P(H_{d}|I)},$$

where *E* is replaced by $\left(\bar{x},ȳ\right)$. For examples where the within-source variance is not known, the sample variances of **x** and **y** will also be included in the representation.

Denote the common mean of the measurements under the prosecution proposition by θ_1_ = θ_2_ = θ. The likelihood ratio V is given by Equation (7). This may be rewritten as

(9) $$\text{V}=\frac{\int f(\bar{x}|\theta )f(\bar{y}|\theta )f(\theta )\text{d}\theta }{\int f(\bar{x}|\theta_{1})f(\theta_{1})\text{d}\theta_{1}\int f(\bar{y}|\theta_{2})f(\theta_{2})\text{d}\theta_{2}},$$

where the dependence on *I* has been suppressed for ease of notation. The analytical form of this likelihood ratio, given the independence and Normality assumptions detailed above, is given by Lindley (1977). The density functions $f(\bar{x}$∣θ) and *f*(ȳ∣θ) are taken to be density functions of a Normal distribution. Note that when the prosecution proposition is chosen the random variables $\bar{X}$ and Ȳ, of which $\bar{x}$ and ȳ are realizations, are conditionally independent, conditional on θ. They are independent if it is known they are from the same source. The distributions associated with these density functions are termed the within-source distributions, because they account for the within-source variability. The distribution associated with the density function *f*(θ) is termed the between-source distribution because it accounts for between-source variability, and it is a prior distribution for θ. The use of a between-source distribution allows the rarity of the data **x** and **y** to be taken into account when assessing the strength of the evidence; see Equation (13) in the Appendix for an example. Information to assist with the estimation of the prior distribution is contained in the training set. If the control and recovered samples have similar means, and the mean is unusual, then the strength of evidence supporting the proposition that the samples are from the same source should be stronger than if the mean is relatively common.

A solution to this problem of the comparison of sources in the case where the measurements are univariate and are assumed to be independent and Normally distributed was developed by Lindley (1977). Some details are given in the Appendix; see Equations (12) and (13) in the Appendix. Denote the *m* measurements on the control sample by **x** = (*x*_1_, …, *x*_*m*_) and the *n* measurements on the recovered sample by **y** = (*y*_1_, …*y*_*n*_). The corresponding means of each of these samples are denoted $\bar{x}$ and ȳ. The two propositions to be considered are at the source level and are:

- *H*_*p*_: the control and recovered sample are from the same source.
- *H*_*d*_: the control and recovered sample are from different sources.

Lindley's solution assumes that the means $\bar{x}$ and ȳ of the control and recovered samples are sample means of data, whose corresponding random variables have Normal distributions with means θ_1_ (control) and θ_2_ (recovered), respectively, and variances σ^2^/*m* (control) and σ^2^/*n* (recovered). The variance σ^2^ is a within-group (e.g., within window) variance. The means θ_1_ and θ_2_ are the means of the groups associated with **x** and **y** in the terminology of hierarchical data. Variability between groups has also to be considered. This is done with consideration of the variation in the group means. The two means θ_1_ and θ_2_ are also assumed to be realizations of a random variable which is Normally distributed, this time with mean μ and variance τ^2^. At present the variances σ^2^ and τ^2^ are assumed known. Also, the within-group variance σ^2^ is assumed constant within groups. An expression for the likelihood ratio if the between-group distribution is not Normal but is represented with a general distribution *p*(·), with second derivative *p*″(·) is given by Equation (14) in Appendix.

An extension using kernel density estimation has been derived to allow for a general non-Normal between-group distribution Equation (15) in Appendix. Checks of the distributional assumptions and estimation of hyperparameters are made using a training set of groups which are assumed to be a random sample of groups (sources) from some relevant population. Later work (e.g., Bozza et al., 2008 with an extension to multivariate data, Equation 24 in Appendix) relaxes the assumption that σ^2^ and τ^2^ are known.

The likelihood ratio can be used to assess evidence in a criminal trial and hence is a solution to the comparison of sources problem; Lindley (1977).

This approach for evidence evaluation based on the likelihood ratio is different from an approach based on hypothesis testing. The likelihood ratio approach has many advantages; a discussion of these can be seen in Aitken and Stoney (1991) and Aitken and Taroni (2004). One such advantage is that the likelihood ratio has no dependence on an arbitrary cut off point (e.g., 5% significance). Another advantage is that the use of a likelihood ratio reduces the risk that a transposition of the conditional probabilities (also known as the prosecutor's fallacy) occurs, a transposition which confuses the probability of finding the evidence on an innocent person with the probability of the innocence of a person on whom the evidence has been found. In addition, the likelihood ratio provides a method of comparing the likelihood of the evidence under the propositions of both the prosecution and the defense. This guards against potentially misleading situations when the likelihood under only one of these propositions is considered. Finally, an approach based on the likelihood ratio ensures equality of treatment of both propositions. In a procedure based on hypothesis testing, a null hypothesis is assumed true unless sufficient evidence is found to reject it at a pre-specified significance level. Often, the null hypothesis is that of a common source, θ_1_ = θ_2_ in Lindley's example. This is the prosecution proposition. Thus the burden of proof is placed on the defense to put forward sufficient evidence to enable rejection of the prosecution proposition, contrary to the dictum of “proof beyond reasonable doubt.” The prosecution need prove nothing.

#### 3.1.2. The likelihood ratio approach for other forms of continuous evidential data, including multivariate data

Later work on evidence evaluation has extended the work done in Lindley (1977) to cover other data types, allowing for different forms of the within and between source distributions (Aitken and Lucy, 2004; Aitken et al., 2006, 2007a). In Bozza et al. (2008) and Alberink et al. (2013), extensions are given so that the between-source distribution in Equation (9) becomes a function of both the mean and the variance. This allows for variation in the variance of samples from different sources. All of these extensions assume that the *m* measurements **x** are independent and that the *n* measurements **y** are independent. Methods for autocorrelated data types, such as measurements associated with drug traces on banknotes are described in Wilson et al. (2014, 2015).

For multivariate measurements which are independent and which have a multivariate Normal distribution the analytical form is derived in Aitken and Lucy (2004). The likelihood ratio is given for two forms of the distribution of the mean between sources. The first form assumes multivariate Normality, and the second form uses nonparametric kernel density estimation. The within-source variance is assumed constant over all sources.

When there are several variables graphical models may be used to reduce the number of parameters needing to be estimated. The kernel density approach given in Aitken and Lucy (2004) can then be used to calculate likelihood ratios for the subsets of variables as indicated by the graphical models. The graphical model considers partial correlations amongst the variables and partitions these variables into overlapping subsets known as *cliques*. The overall distribution may then be represented as a function of the distributions over the cliques. These clique distributions have very few variables each (e.g., one, two or three; and the overall likelihood ratio is then a product of likelihood ratios which are based on one-, two- or three-dimensional data Aitken et al., 2007). Such a process for the reduction of dimension is necessary to avoid the curse of dimensionality whereby very large data sets are needed for the estimation of parameters in a multi-dimensional parameter set.

In Aitken et al. (2006) the multivariate methods used in Aitken and Lucy (2004) assuming Normality are extended further to allow for another level of variance (e.g., measurement error) to be taken into account, giving a three-level model. A model assuming an exponential distribution for between-sources in a three-level model is assumed in Aitken et al. (2007a) and the analytical form of the likelihood ratio is derived. Variation between the means of samples from different sources, variation between the means of different samples taken from the same source and variation within repeated measurements on the same sample are taken into account.

Relaxation of the assumption that samples from different sources will have the same variance means that an analytical solution is not available. Measurements are assumed multivariate and independently Normally distributed as before but the between-source (prior) distribution is taken to be the product of a multivariate Normal distribution (for the mean of the between-source distribution) and an inverse Wishart distribution (for the covariance of the between-source distribution). In this way, variation of covariances, as well as means, between different sources is taken into account. An analytical form of the likelihood ratio is not available so Markov chain Monte Carlo (MCMC) methods are used to estimate it (Bozza et al., 2008) (Equation 24 in the Appendix).

A similar approach to Bozza et al. (2008) for the evaluation of the likelihood ratio for the comparison of sources problem is used by Alberink et al. (2013) in that variation in the variance parameter between sources is modeled as well as variation in the mean parameter, although in Alberink et al. (2013) the data are univariate. As with all of the other approaches discussed, the within-source distribution is Normal, and the data are assumed independent. There are two main extensions seen in Alberink et al. (2013). The first is that three different distributions are used for the between-source distribution. One is the univariate equivalent of the between-source distribution used in Bozza et al. (2008) (a semi-conjugate prior), one is a non-informative prior, proportional to the inverse of the variance, and one is the conjugate prior distribution seen on p. 74 of Gelman et al. (2004). This conjugate prior distribution gives a between-source distribution for the parameter (μ, σ^2^), denoting group mean and variance, of

$$\begin{array}{l} \mu \sim N(\mu_{0},\sigma^{2}/\kappa_{0})\\ \sigma^{2}\sim \text{Inv-}\chi^{2}(\nu_{0},\sigma_{0}^{2}) \end{array}$$

where μ_0_, κ_0_, ν_0_ and $\sigma_{0}^{2}$ are hyperparameters to be estimated and the notation Inv-χ^2^ corresponds to a scaled inverse chi-squared distribution. The difference between this and the univariate equivalent of the between-source distribution used in Bozza et al. (2008) is that the variance of the parameter μ is proportional to σ^2^. An analytical form of the likelihood ratio for the two cases when the between-source distribution is given by the non-informative prior and when the between-source distribution is given by the conjugate prior (Alberink et al., 2013) who also show that no analytic solution exists if a semi-conjugate prior is used. (See Equations (16,17) in the Appendix.)

As in Bozza et al. (2008), Alberink et al. (2013) use MCMC methods to evaluate the likelihood ratio when the between-source distribution is given by the semi-conjugate prior, although there are differences in the implementation, leading to the second main extension. Alberink et al. (2013) use prior distributions on the hyperparameters of the between-source distribution and then combine these prior distributions with training data to obtain a posterior distribution for the hyperparameters, conditional on the training data. All of the other methods discussed estimate the parameters of the between-source distribution directly from the training data using summary statistics. The methods used in Alberink et al. (2013) allow for a Bayesian approach for the estimation of the between-source distribution. One disadvantage of this approach is that the method for estimating the likelihood ratio used in Bozza et al. (2008) is no longer feasible because, instead of having a known analytic form for the between-source density function, draws from the between-source distribution are obtained using MCMC methods. Monte Carlo integration is used by Alberink et al. (2013) to estimate the likelihood ratio.

All of the literature discussed in sections 3.1.1 and 3.1.2 evaluates likelihood ratios for continuous evidential data. There are some common assumptions. All assume that measurements are independent and that the within-source distribution is Normal (univariate or multivariate). Constant variation between sources of the within-source distribution is assumed by Lindley (1977), Aitken and Lucy (2004), Aitken et al. (2007) and Aitken et al. (2006). This assumption is relaxed by Bozza et al. (2008) and Alberink et al. (2013), allowing the variance to vary between sources. A Bayesian approach is used by Alberink et al. (2013) to obtain the parameters of the between-source distribution.

Methods for the evaluation of continuous, autocorrelated data are described in Wilson et al. (2014) and Wilson et al. (2015). The data used for illustration are the quantities of drugs on banknotes where quantities on adjacent notes cannot be considered independent. Some work has also been done on the evaluation of evidence for discrete data, particularly in the field of DNA profiling (Buckleton et al., 2005) and more recently on data relating to clicks in speech (Aitken and Gold, 2013) and the presence or absence (binary data) of striation marks for screwdrivers (Aitken and Huang, 2015).

### 3.2. Discrimination

Forensic scientists are not only interested in comparisons of two pieces of evidence, such as control and recovered evidence, under different propositions, that of same source vs. that of different source, without attention being paid to the identity of the source. There is also interest in the source of one piece of evidence. The support of the evidence for a proposition of source is of interest. The problem concerns the determination of whether a sample of data is more likely to be from one population (source) or another. Of course, such a determination is the concern of the fact-finder. The scientist is concerned with the probability of the measurements on the evidential material if the material came from one source or if it came from another. If there are more than two possible sources, then prior probabilities, that is, probabilities for each source under consideration before the material is examined, are needed in order to obtain a likelihood ratio. In this problem there is only one set of evidential data compared with the two sets (control and recovered) in the comparison problem. The aim is to assist the decision-maker as to the population of origin of the evidential data. This is a problem of *discrimination*, as distinct from a problem of *comparison*.

An example of the use of likelihood ratios in a problem of this sort can be seen in Zadora et al. (2010) which looks at the discrimination of glass samples and in Wilson et al. (2014, 2015) which considers discrimination between banknotes assocated with a person associated with criminal activity and banknotes associated with a person not associated with criminal activity. As with the problem of comparison of sources, the likelihood ratio alone cannot determine whether a set of data is more likely from one population or another; it must be considered in conjunction with the prior odds. The derivation of the likelihood ratio for such discrimination problems is discussed in Taroni et al. (2010) (Chapter 8). The likelihood ratio for a set of evidence consisting of *n* measurements, **z** = (*z*_1_, …, *z*_*n*_), under two propositions, *H*_*p*_ and *H*_*d*_, is considered.^4^ The two propositions are given by

- *H*_*p*_ : data **z** are from population 1, and
- *H*_*d*_ : data **z** are from population 2.

The likelihood ratio V for the discrimination problem, where *I* is the background information as usual, is given in Taroni et al. (2010) by

(10) $$\text{V}=\frac{f(z|H_{p},I)}{f(z|H_{d},I)}.$$

This expression can be compared with Equation (7) and the comparison problem. In the comparison context, the joint density function of control and recovered data is considered. In the discrimination problem, two (or more) possible sources (populations) are identified.

Assume as for the comparison problem that the data are hierarchical and that there are two possible sources. The probability density function of groups of data from source *i* is parameterized by θ_*i*_, *i* = 1, 2 (possibly multivariate). If the value of θ_*i*_ (for *i* ∈ {1, 2}) varies between different groups in population *i* then by conditioning on θ_1_ in the numerator and θ_2_ in the denominator, the likelihood ratio V can be written

(11) $$\text{V}=\frac{\int f(z|\theta_{1})f(\theta_{1})\text{d}\theta_{1}}{\int f(z|\theta_{2})f(\theta_{2})\text{d}\theta_{2}}.$$

The probability density function *f*(θ_*i*_) models the variability of the parameter θ_*i*_ between groups in population *i*, and is termed the between-group density function (the associated distribution function will be termed the between-group distribution function). This is analogous to the between-source distribution used to model variability between sources in the comparison of sources problem. Similarly, the density function *f*(**z** ∣ θ_*i*_) is termed the within-group density function (with the associated distribution function termed the within-group distribution function).

Using this formulation for the likelihood ratio, the methods discussed previously for the evaluation of the likelihood ratio for the comparison of sources problem can be adapted to evaluate the value of evidence for discrimination problems. The limitations and assumptions of these methods still apply.

In the context of discrimination, training data are a random sample of groups from each or both of the sources. Variation is between groups within each of the sources. There is an abuse of terminology here. In the comparison problem with the proposition of common source, the control and recovered evidence are deemed to be from the same source but without specification of the source. The source is a member of a population of sources. In the discrimination problem, support for a particular source is assessed. The distinction between comparison and discrimination problems is emphasized in Zadora et al. (2014) where the two problems are discussed in different chapters (and note that discrimination is there noted as classification).

### 3.3. Score-based models

Return now to consideration of the problem of comparison of sources with a *p*-dimensional control measurement **x** = (*x*_1_, …, *x*_*p*_) and a *p*-dimensional recovered measurement **y** = (*y*_1_, …, *y*_*p*_). For those occasions when a feature-based model is not tractable (e.g., multidimensional binary data), the distance *d*(**x**, **y**), known as a *score* can be used instead. The value of the evidence is then

$$\text{V}=\frac{f(d(x,y)|H_{p},I)}{f(d(x,y)|H_{d},I)}.$$

Rarity is not considered. Inference may then continue as before but using the score, which is univariate, as the statistic of interest. Score-based approaches estimate the probability distribution function of a calculated score. Score-based approaches have been used for handwriting (Hepler et al., 2012) and speech recognition (Brümmer and Du Preez, 2006; Gonzalez-Rodriguez et al., 2006; Morrison, 2011). Score-based methods do not require the distributional assumptions (such as within-source Normality) needed to fit the models described above but do still require a function to be chosen to model the probability distribution function of the score.

There are various distance measures that may be used. Three examples are

- Euclidean: $d=\sqrt{\overset{p}{\underset{i=1}{\sum }}{\left(x_{i}-y_{i}\right)}^{2}}$;
- Manhattan: $d=\overset{p}{\underset{i=1}{\sum }}|x_{i}-y_{i}|$;
- Pearson correlation distance: 100(1 − *r*)/2 with
  $$r=\frac{\sum_{i=1}^{p}\left(x_{i}-\bar{x})(y_{i}-\bar{y}\right)}{\sqrt{\sum_{i=1}^{p}{\left(x_{i}-\bar{x}\right)}^{2}\sum_{i=1}^{p}{\left(y_{i}-\bar{y}\right)}^{2}}}.$$

Other examples are available in SAILR. For multiple control and recovered data **x**_*i*_, *i* = 1, …, *m* and **y**_*i*_, *i* = 1, …, *n*, respectively, pairwise score measurements or means can be used.

For the calculation of score-based likelihood ratios, distributions of scores of same-source comparisons and of different-source comparisons are required. Determination of the same-source distribution can be made by comparing every measurement in a training set **z** with every other measurement within its own source except with itself for which the distance is zero. For the different-source distribution, every measurement is compared with all measurements from other sources. These results may then be used to estimate the distributions of same-source and between-source comparisons. The distributions can be represented initially by histograms. They may then be smoothed with a kernel density estimation or an appropriate parametric distribution. The current choice of parametric distribution in SAILR is a Gamma distribution or a Weibull distribution. The chosen distribution functions, one for same-source comparisons and one for different-source comparisons, then can be used to determine the density calculation of the evidence score for both distributions and hence calculate a likelihood ratio.

### 3.4. Comparison of feature-based and score-based models

Models for discrimination and for comparison that use the original data are feature-based models. The models discussed in sections 3.1 and 3.2 are all feature-based. Feature-based multivariate Normal models compare the probability of observing the evidence given that the evidential samples (control and recovered) measured, and compared, come from the same source or come from different sources. In contrast, the score-based model compares the probability of observing the pairwise similarity between two samples (control and recovered) given that they come from the same source with the probability of the pairwise similarity given that the samples come from different sources. A comparison of the performances of score-based and frequency-based likelihood ratios for forensic MDMA comparisons is given in Bolck et al. (2015).

The benefits and shortcomings of both methods are given by Bolck et al. (2015) as:

- Feature-based benefits:
  - Original data dimensionality preserved; no information loss.
  - Rarity and similarity of the features relate directly to the magnitude of the likelihood ratio.

- Feature-based shortcomings:
  - Covariance estimation is difficult when limited data are available relative to the dimensionality of the variables.
  - The feature-based method is often less robust than the score-based model when there are limited population samples.

- Score-based benefits:
  - Covariance estimation between sources is possible with few samples available.
  - The method is robust and able to be generalized to new samples.

- Score-based shortcomings:
  - There is a loss of information because of a reduction of dimensionality.
  - The value of the likelihood ratio is based on the similarity of pairwise scores rather than the similarity and rarity of features.

### 3.5. Summary of feature-based models

References for details of a selection of feature-based two-level models with within-group measurements independent and Normally distributed are listed here. Equation numbers are given for models for which further details are given in the Appendix.

- Univariate:
  - Within-group Normal,
    Between-group Normal for between-group mean (assume within-group variance known)
    (Lindley, 1977, see Equations 12, 13 in the Appendix).
  - Within-group Normal,
    Between-group Taylor expansion for between-group mean (assume within-group variance known)
    (Lindley, 1977, see Equation 14 in the Appendix).
  - Within-group Normal,
    Between-group kernel for between-group mean (assume within-group variance known),
    (Aitken and Taroni, 2004, see Equation 14 in the Appendix).
  - Within-group Normal,
    Between-group distribution:
    (a) Normal distribution - semi-conjugate prior,
    (b) Non-informative prior, proportional to the inverse of the variance,
    (c) Conjugate prior - Normal, scaled inverse chi-squared (Alberink et al., 2013, see Equations 15, 16 in the Appendix).

- Bivariate:
  Numerator (predictive distribution) (Bernardo and Smith, 1994),
  Denominator (kernel),
  (Evett et al., 1987, see Equation 18 in the Appendix).

- Multivariate, within-group measurements independent and Normally distributed
  - Within-group Normal,
    Between-group kernel for distribution of group means, Within-group variance assumed common and estimated from training data, (Aitken and Lucy, 2004, 4.1, see Equation 19 in the Appendix).
  - Within-group Normal
    Between-group Normal for distribution of group means, Inverse Wishart for the covariance of within-source distribution, (Bozza et al., 2008, see Equation 24 in the Appendix).
  - With graphical models:
    See section 3.1.1; Aitken et al. (2007).
  - In the presence of zeros, that is when no measurement of a specific characteristic has been made on certain members of the control data set, the recovered data set or the training data set: both Normal and kernel between-group distributions considered. Estimation of covariance matrices by imputation and by available cases (Zadora et al., 2010).
  - In addition, when within-group measurements are autocorrelated and Normally distributed (see Wilson et al., 2014, 2015).

## 4. Model performance

Model performance for the comparison problem is assessed with a training set and associated data **z** as discussed in section 2.6. If possible, another set, known as a *validation* set could be used. The training set and validation set should both comprise several sources of data from a relevant population. Within each source, measurements are taken on each of several items. The source of each member of the two sets is known. Models and parameters can be fitted using the training set. The performance can be assessed using the validation set. Thus when a method for comparison or discrimination is tested using members of the data set it is known if the correct answer is given. In the absence of a validation set, the performance can be assessed through a second use of the training set (e.g., with a leaving-one-out method). Validation enables the provision of measures of performance based on calculated likelihood ratios.

For a comparison of two members of the validation (or training) set a likelihood ratio is calculated. There are two conclusions that may be drawn by the fact-finder: they are from the same source or they are not from the same source. If the likelihood ratio is greater than 1, then this is support for the proposition of a common source for the two members of the validation (training) set being compared. If they are truly from the same source then this is counted as a correct result. Similarly, if its value is less than 1, then this is support for the proposition of different sources for the two members of the validation (training) set being compared. If they are truly from different sources then this is counted as a correct result. However, if the two members have a value for the likelihood ratio of greater than 1 when they are from different sources, this is an incorrect result and the result is known as a *false positive*. Similarly, if the two members have a value for the likelihood ratio of less than 1 when they are from the same source, this is an incorrect result and the result is known as a *false negative*.

For discrimination with two groups, say *A* and *B*, the member of the data set may be classified by the fact-finder as belonging to group *A* or to group *B*. False positives and false negatives can be defined in a manner analogous to that of the comparison procedure. A likelihood ratio is calculated. If its value is greater than 1, then this is support for the proposition that the member of the training set belongs to group *A*, say. If the member is truly from group *A* then this is counted as a correct result. Similarly, if its value is less than 1, then this is support for the proposition that the member is from group *B*. If it is truly from group *B*, then this is counted as a correct result. However, if the member has a value for the likelihood ratio of greater than 1 when it is from group *B*, this is an incorrect result and the result is a false positive, say. Similarly, if the member has a value for the likelihood ratio of less than 1 when it is from group *A*, this is an incorrect result and the result is a false negative.

For both comparison and discrimination problems, the strength of the support is measured by the value of the likelihood ratio. As noted in section 2.3 if the logarithm is taken this is known as the weight of evidence. Given the existence of a validation (training) set it is possible to measure the performance of a method for comparison or discrimination as the correct answer is known. It is not possible to assess the result in an individual case; the correct answer in an individual case is not known.

The likelihood ratio, or a function of it such as the logarithm, has been shown by Good, 1989a,b (section 2.2) to provide the best (only) value of the evidence. Attempts to express the uncertainty associated with this assessment (e.g. with a confidence interval) are attempts to put a probability on a probability and should not be done (Taroni et al., 2016). This view is not universally agreed, see discussion issues of *Law, Probability and Risk* (2016, volume 15, issue 1) and *Science and Justice* (2017, volume 56). Note also the quote from Kaye (1979) in section 2: “It thus supplies the jurors with as precise and accurate an illustration of the probative force of the quantitative data as the mathematical theory of probability can provide”. It is not necessary to provide an interval estimate.

There are several measures of performance.

- *The percentage of false positives and of false negatives amongst all the comparisons or discriminations tested*. Often, in a criminal case, one of the propositions is associated with the prosecution, hence the notation *H*_*p*_, and other is associated with the defense, with the notation *H*_*d*_. In such a circumstance, the burden of proof lies with the prosecution. It is a more serious error to support the prosecution proposition wrongly than to support the defense proposition wrongly. Let support for the prosecution proposition be known as a positive result. Thus, when considering the performance of a test, it is better to choose a test in which there is a low false positive rate and a high false negative rate rather than one in which there is a high false positive rate and low false negative rate. Ideally, zero false positive and zero false negative results are best but such an ideal is rarely achieved.
- *A Tippett plot*. See Evett and Buckleton (1996) and Tippett et al. (1968). This is a graphical measure of rates of misleading evidence for comparisons. It is the complement of empirical cumulative distribution functions for same-source and different-source comparisons. The plots come in pairs, one for same-source comparisons and one for different-source comparisons. The *log*(*LR*) is plotted on the *x*-axis and, for a particular value *x*_0_ of the *log*(*LR*), the *y*-axis is the relative frequency of the number of comparisons greater than *x*_0_. For same-source comparisons, it is to be hoped that all *log*(*LR*) values are greater than 0. Thus for *x* < 0, it is hoped the corresponding value on the *y*-axis will be 1 (or 100%). Similarly, for different-source comparisons, it is to be hoped that all *log*(*LR*) values are less than 0. Thus for *x* > 0, it is hoped the corresponding value on the *y*-axis will be 0 (or 0%).
  The vertical distance from the intersection of the same-source plot with the line *log*(*LR*) = 0 and the line *y* = 1(100%) is the rate of misleading evidence for same-source comparisons, the proportion of same-source comparisons that have a value of *log*(*LR*) < 0(*LR* = 1). The vertical distance from the intersection of the different-source plot with the line *log*(*LR*) = 0 and the line *y* = 0(0%) is the rate of misleading evidence for different-source comparisons, the proportion of different-source comparisons that have a value of *log*(*LR*) > 0 (*LR* = 1).
- *Detection error trade-off (DET) curves*. See Meuwly et al. (2017). A detection error trade-off (DET) plot is a 2-dimensional graphical representation in which the proportion of false positives is plotted as a function of the proportion of false negatives. The closer the curves to the coordinate origin, the better are the discriminating capabilities of the method. The intersection of a DET curve with the main diagonal of the DET plot marks the Equal Error Rate (EER) which is the point when the proportions of false positives and false negatives are equal.
- *Empirical cross-entropy*. See Meuwly et al. (2017), Ramos et al. (2013) and Ramos and Gonzalez-Rodriguez (2013).
  The performance of probabilistic assessments has been addressed by *strictly proper scoring rules* (SPSR). Consider two propositions about a parameter θ, one that θ = θ_*p*_ and one that θ = θ_*d*_, with Pr(θ = θ_*p*_) = 1 − Pr(θ = θ_*d*_) For evidence evaluation, the *logarithmic* SPSR is used and defined as
  $$\begin{array}{l} C(\mathrm{Pr}(\theta_{p}|I),\theta )=-\log_{2}(\mathrm{Pr}(\theta_{p}|I))\text{ if }\theta =\theta_{p},\\ \;=-\log_{2}(1-\mathrm{Pr}(\theta_{d}|I))\text{ if }\theta =\theta_{d}, \end{array}$$
  The measure of accuracy for evidence evaluation based on the SPSR is a weighted average value of the logarithmic scoring rule, and is known as the *empirical cross-entropy* (*ECE*):
  $$\begin{array}{l} ECE=-\frac{\mathrm{Pr}(\theta_{p}|I)}{N_{p}}\sum_{\theta_{\left(i\right)}=\theta_{p}}\log_{2}\mathrm{Pr}(\theta_{p}|E_{i},I)\\ \;-\frac{\mathrm{Pr}(\theta_{d}|I)}{N_{d}}\sum_{\theta_{\left(j\right)}=\theta_{d}}\log_{2}\mathrm{Pr}(\theta_{d}|E_{j},I)\\ \;=\frac{\mathrm{Pr}(\theta_{p}|I)}{N_{p}}\sum_{\theta_{\left(i\right)}=\theta_{p}}\log_{2}(1+\frac{1}{LR_{i}\times O(\theta_{p})})\\ \;+\frac{\mathrm{Pr}(\theta_{d}|I)}{N_{d}}\sum_{\theta_{\left(j\right)}=\theta_{d}}\log_{2}(1+LR_{j}\times O(\theta_{p})), \end{array}$$
  where *LR*_*i*_(*LR*_*j*_) is the likelihood ratio for the *i*-th (*j*-th)*E*_*i*_ (*E*_*j*_) piece of evidence where θ = θ_*i*_(θ_*j*_), respectively, and *O*(θ_*p*_) denotes the prior odds Pr(*H*_*p*_)/*Pr*(*H*_*d*_). For the discrimination problem with two sources, the parameters θ_*p*_ and θ_*d*_ represent the parameters of the two sources. For the comparison problem θ_*p*_ represents same-source comparisons and θ_*d*_ represents different-source comparisons in the validation dataset.

This measure tends to indicate better performance when the likelihood ratio leads to the correct decision. The numerical value will be lower as the performance increases. The ECE can be represented as an ECE-plot, showing its value for a certain range of priors.

### 4.1. Conclusion

The development of methods for the evaluation of evidence for frequency-based continuous two-level models is described from the hierarchical model for univariate continuous data developed by Lindley (1977) to multivariate models with unknown means and covariances (Bozza et al., 2008). This development is of interest in its own right as a compilation of some thirty years of development. However, it also provides a background to the development of the SAILR package, a package which extends these ideas to include score-based models.

Formulae for many of these are given in the Appendix and may also be found in many books on the subject (e.g., Aitken and Taroni, 2004; Zadora et al., 2014).

There is much more that can be reviewed. References for some of the omissions of this paper are given here. It is hoped they are useful. There have been few papers on models for discrete data; see Aitken and Gold (2013) for an example. Score-based models have received a lot of attention recently and are included in SAILR; see Bolck et al. (2015) for examples. Graphical models provide an approach for a reduction in the dimensionality of multivariate problems; see Zadora et al. (2014) for examples.

## Author contributions

The author confirms being the sole contributor of this work and approved it for publication.

### Conflict of interest statement

The author declares that the research was conducted in the absence of any commercial or financial relationships that could be construed as a potential conflict of interest.

## Footnotes

^1^Other writers use the term *hypothesis* (see section 2.7). The term *proposition* will he used except when there is an explicit need for the term *hypothesis*; see, for example, section 3.1

^2^In ENFSI guidelines ENFSI (2015) “findings” are distinguished from “evidence.” “Findings are the result of observations, measurements and classification that are made on items of interest.” “[E]vidence refers to outcomes of forensic examinations (findings) that, at a later point, may be used by legal decision-makers in a court of law to reach a reasoned belief about a proposition.” However, the word “evidence” will be used in this document to refer to both situations for ease of nomenclature.

^3^The use of **x** and **y** here is not to be confused with the use of *x* = Pr(*E* ∣ *H*_*p*_) and *y* = *Pr*(*E* ∣ *H*_*d*_) in section 2.2.

^4^Note the change of use of notation. In this section, **z** refers to evidential data and not to training data.
