# Supplementary material for: Bayesian Hierarchical Random Effects Models in Forensic Science
Source: Front Genet. 2018 Apr 16;9:126. doi: 10.3389/fgene.2018.00126 (PMC5911710; doi:10.3389/fgene.2018.00126)
Supplement: Supplementary file 1 [file DataSheet1.pdf]

Bayesian hierarchical random effects models in forensic science

C.G.G. Aitken [1,*]

[1,*] *School of Mathematics and Maxwell Institute, The University of Edinburgh, Peter Guthrie Tait Road, Edinburgh, EH9 3FD, UK*

Correspondence*:
C.G.G. Aitken
cgga@ed.ac.uk

Appendix: Inventory of frequency-based continuous two-level models

The purpose of the inventory is to illustrate the development of methods for the evaluation of evidence for frequency-based continuous two-level models. The formulae that are given are for the likelihood ratio, denoted V (9), with the purpose of illustrating how rarity and similarity are assessed within the same formula and how uncertainty in means and variances is considered.

There are no derivations of formulae. Source references where the derivations may be found are given in association with each model. Whilst SAILR provides a software package for evaluation of evidence, appropriate R code is also available elsewhere, e.g., Zadora et al. [2014].

General notation for univariate models (Lindley [1977])

Measurements are Normally distributed about the true values with a known, constant variance σ^2. For m measurements $\mathbf{x}_1, \ldots, \mathbf{x}_m$) of a control item, the mean $\bar{X}$ (the random variable corresponding to observation $\bar{x}$ is Normally distributed with mean θ_1 and variance σ^2/m. For n measurements $(y_1, \ldots, y_m)$ of a recovered item, the mean $\bar{Y}$ is Normally distributed with mean θ_2 and variance σ^2/n. If the control and recovered items come from the same source, the prosecution proposition H_p, then $\theta_1 = \theta_2$. If the control and recovered items come from different sources, the defence proposition H_d, then $\theta_1 \neq \theta_2$.

Assume $\theta \sim N(\mu, \tau^2)$ and let

$$a^2 = \frac{1}{m} + \frac{1}{n},\ \sigma_1^2 = \tau^2 + \sigma^2/m,\ \sigma_2^2 = \tau^2 + \sigma^2/n,\ \sigma_3^2 = \tau^2 + \sigma^2/(m+n),$$

and

$$W = (m\bar{X} + n\bar{Y})/(m+n), \quad Z = (\sigma_2^2\bar{X} + \sigma_1^2\bar{Y})/(\sigma_1^2 + \sigma_2^2).$$

Formulae are given below for realisations of these random variables: thus $\bar{X}, \bar{Y}, W$ and Z are replaced by $\bar{x}, \bar{y}, w$ and z

Value of evidence for univariate models

- The distribution of the true values θ is Normal, mean μ and variance τ^2, where τ^2 is assumed known.

$$V = \frac{\sigma_1\sigma_2}{a\sigma\sigma_3} \exp\left\{-\frac{(\bar{x}-\bar{y})^2\tau^2}{a^2\sigma^2(\sigma_1^2+\sigma_2^2)}\right\} \exp\left\{-\frac{(w-\mu)^2}{2\sigma_3^2} + \frac{(z-\mu)^2(\sigma_1^2+\sigma_2^2)}{2\sigma_1^2\sigma_2^2}\right\}. \quad (12)$$

- The between-group standard deviation $\tau \gg \sigma$ such that $\sigma_1^2 = \sigma_2^2 = \sigma_3^2 = \tau^2$ and $z = (\bar{x} + \bar{y})/2$ and $m = n = 1$ without loss of generality then

$$V = \frac{\tau}{\sigma\sqrt{2}} \exp\left\{ -\frac{(\bar{x} - \bar{y})^2}{4\sigma^2} \right\} \exp\left\{ \frac{(z - \mu)^2}{2\tau^2} \right\}. \tag{13}$$

The term $(\bar{x} - \bar{y})^2/4\sigma^2$ is a measure of similarity. The more similar (closer together) $\bar{x}$ and $\bar{y}$ are, the smaller $(\bar{x} - \bar{y})^2/4\sigma^2$ is and hence the larger the term $\exp\left\{ -(\bar{x} - \bar{y})^2/4\sigma^2 \right\}$ is (note the negative sign) and hence the larger V is. The term $(z - \mu)^2/2\tau^2$ is a measure of rarity. The overall mean of the population from which the measurements are assumed to have come is μ. The mean z of the control mean $\bar{x}$ and recovered mean $\bar{y}$, weighted by their variances so that the mean with the smaller variance is given the larger weight is compared with the overall mean. The further z is from μ, the larger the term $(z - \mu)^2/2\tau^2$ is and hence the larger the term $\exp(z - \mu)^2/2\tau^2$ is (note the implicit positive sign) and hence the larger V is.

- The between-group distribution is not Normal but is represented with a general distribution $p(\cdot)$, with second derivative $p''(\cdot)$ then

$$V = \frac{1}{a\sigma\sqrt{2\pi}} \exp\left\{ -\frac{(\bar{x} - \bar{y})^2}{2a^2\sigma^2} \right\} \frac{p(w) + \frac{1}{2}p''(w)\,\sigma^2/(m+n)}{\{p(x) + \frac{1}{2}p''(\bar{x})\sigma^2/m\}\{p(\bar{y}) + \frac{1}{2}p''(y)\sigma^2/n\}}. \tag{14}$$

- The between-group distribution is represented by a kernel density estimate (Aitken and Taroni [2004], p. 338). Consider background data of the form $\{z_{ij}, i = 1, \ldots, k; j = 1, \ldots, l\}$ where k is the number of groups and l is the number of members of each group, assumed constant amongst groups. Let $\bar{z}_i$ denote the mean of the i-th group and $\bar{z}$ the overall mean. The within-group variance is then estimated by

$$\hat{\sigma}^2 = \sum_{i=1}^{k} \sum_{j=1}^{l} (z_{ij} - \bar{z}_i)^2/(kl - k)$$

and the between-group variance τ^2 by

$$s^2 = \sum_{i=1}^{k} (\bar{z}_i - \bar{z})^2/(k-1) - \hat{\sigma}^2/l.$$

$$V = \frac{K \ \exp\{-\frac{(\bar{x}-\bar{y})^2}{2a^2\sigma^2}\} \sum_{i=1}^{k} \ \exp\{-\frac{(m+n)(w-z_i)^2}{2[\sigma^2+(m+n)s^2\lambda^2]}\}}{\sum_{i=1}^{k} \ \exp\{-\frac{m(\bar{x}-z_i)^2}{2(\sigma^2+ms^2\lambda^2)}\} \sum_{i=1}^{k} \ \exp\{-\frac{n(\bar{y}-z_i)^2}{2(\sigma^2+ns^2\lambda^2)}\}} \tag{15}$$

where

$$K = \frac{k\sqrt{(m+n)}\sqrt{(\sigma^2 + ms^2\lambda^2)}\sqrt{(\sigma^2 + ns^2\lambda^2)}}{a\sigma\sqrt{(mn)}\sqrt{\{\sigma^2 + (m+n)s^2\lambda^2\}}}.$$

- The distribution of the true values θ is Normal, mean μ and variance τ^2, where τ^2 is not assumed known (Alberink et al. [2013]). Conjugate priors are chosen for θ and σ^2. The prior distribution for θ, or more rigorously, $\theta \mid \tau^2$ is $N(\mu, \tau^2/\kappa_0)$, for parameters μ and κ_0. In this situation, a prior is introduced for τ, which is such that $\nu_0\tau_0^2/\tau^2 \sim \chi^2(\nu_0)$ for parameters ν_0 and τ_0. Formulaically, the

joint prior is

$$p_2(\theta,\sigma^2) = c_2^{-1}(\sigma^2)^{-(\nu_0+3)/2}\exp\Big(-\frac{1}{2}\sigma^{-2}(\nu_0\tau_0^2+\kappa_0(\tau-\tau_0)^2)\Big),$$

with c_2 a normalising constant. Let

$$\rho_0 = \nu_0\tau_0^2, \rho_k = \nu_0\tau_0^2 + n_k s_k^2 + \frac{\kappa_0 n_k}{k_0+n_k}(\bar{x}_k-\mu_0)^2,$$

with $k = 1,2$, and

$$\rho_{1,2} = \nu_0\tau_0^2 + \sum_{k=1}^{2} n_k s_k^2 + \sum_{k=1}^{2}\frac{\kappa_0 n_k}{k_0+n_k}(\bar{x}_k-\mu_0)^2 + \frac{n_1 n_2}{\kappa_0+n}(\bar{x}_1-\bar{x}_2)^2.$$

The likelihood ratio is then

$$\begin{aligned} LR &= \frac{\Gamma(\nu_0/2)\Gamma((\nu_0+n)/2)}{\Gamma((\nu_0+n_1)/2)\Gamma((\nu_0+n_2)/2)}\Big(\frac{(\kappa_0+n_1)(\kappa_0+n_2)}{\kappa_0(\kappa_0+n)}\Big) \\ &\quad\times\Big(\frac{\rho_1}{\rho_{1,2}}\Big)^{n_1/2}\Big(\frac{\rho_2}{\rho_{1,2}}\Big)^{n_2/2}\Big(\frac{\rho_1\rho_2}{\rho_0\rho_{1,2}}\Big)^{\nu_0/2}, \end{aligned} \quad (16)$$

[Alberink et al., 2013].

- A semi-conjugate prior can be chosen for θ and σ^2 (Alberink et al. [2013]) such that $\theta \sim N(\mu_0,\tau_0^2)$ and σ^2 has an inverse chi-squared distribution with parameters (ν_0,σ_0^2) such that ν_0,σ_0,μ_0 and τ_0 and the mean and variance are statistically independent. Then

$$p_3(\mu,\sigma^2) = c_3^{-1}(\sigma^2)^{-(\nu_0+2)/2}\exp\Big(-\frac{1}{2}(\nu_0\sigma_0^2\sigma^{-2}+\tau_0^{-2}(\mu_0-\mu)^2)\Big) \quad (17)$$

with c_3 the normalising constant, [Alberink et al., 2013]

Value of evidence for multivariate models

- An early approach to the estimation of the likelihood ratio for multivariate data was used in the case of bivariate colour chromaticity co-ordinates for fibres (Evett et al. [1987]). Let $\mathbf{y}$ denote a bivariate vector of complementary chromaticity co-ordinates measured from a fibre found at the crime scene and assumed to come from an article of clothing worn by the criminal. Let $\mathbf{x} = (\mathbf{x_1},\ldots,\mathbf{x_m})$ denote a set of bivariate vectors of complementary chromaticity co-ordinates measured from m fibres taken to be a representative sample from a garment belonging to a suspect. The propositions are H_p : the recovered fibre came from the suspect's garment, and H_d: the recovered fibre came from some other source.

  The numerator of the likelihood ratio is taken to be $f(\mathbf{y} \mid H_p,\mathbf{x})$ and the denominator to be $f(\mathbf{y} \mid H_d)$; see (**??**).

  The measurements were assumed to have distributions $f(\mathbf{y} \mid \mu,\Sigma)$ and $f(\mathbf{x_i} \mid \mu,\Sigma),\ i=1,\ldots,m$ that were bivariate Normal with mean μ and covariance matrix Σ. Vague priors are chosen for μ and Σ:

$$f(\mu \mid \Sigma) \propto c \text{ for } \mu \text{ and } f(\Sigma) \propto \mid\Sigma\mid^{-3/2} \text{ for } \Sigma,$$

where c is a constant, independent of μ. The probability density function for $f(\mathbf{y} \mid \mathbf{x}, H_p)$ is then a bivariate Student density function of the form:

$$\frac{\Gamma(m/2)}{\pi\Gamma((m-2)/2)} \Bigg/ \left\{ \left| \frac{(m-1)(m+1)}{m} S_x \right|^{1/2} \left[1 + (\mathbf{y} - \bar{\mathbf{x}})' \frac{(m-1)(m+1)}{m} S_x^{-1} (\mathbf{y} - \bar{\mathbf{x}}) \right]^{m/2} \right\} \quad (18)$$

where $\bar{\mathbf{x}}$ and S_x are the sample mean and covariance matrix, respectively, for the measurements (Aitchison and Dunsmore [1975], Aitchison et al. [1977]). The denominator $f(\mathbf{y} \mid H_d)$ is taken as a kernel density estimate. Further work on likelihood ratios for fibre evidence of complementary chromaticity co-ordinates is described in Wakefield et al. [1991].

- Likelihood ratio with the assumption of constant within-source variation and between-source normality; see (Aitken and Lucy [2004]).

Let Ω denote a population of p characteristics of items of a particular evidential type. Background data are measurements of these characteristics on a random sample of m members from Ω with $n(\geq 2)$ replicate measurements on each of the m members. The background data are denoted as $\mathbf{x}_{ij} = (x_{ij1}, \ldots, x_{ijp})^T, i = 1, \ldots, m, j = 1, \ldots, n$ with

$$\bar{\mathbf{x}}_i = \frac{1}{n} \sum_{j=1}^{n} \mathbf{x}_{ij}.$$

The control and recovered measurements are denoted by $\{\mathbf{y}_l\} = (\mathbf{y}_{lj}, j = 1, \ldots, n_l, l = 1, 2)$ where $\mathbf{y}_{lj} = (y_{lj1}, \ldots, x_{ljp})^T$, with

$$\bar{\mathbf{y}}_l = \frac{1}{n_l} \sum_{j=1}^{n_l} \mathbf{y}_{lj}.$$

For within-source variation, the mean vector within source i is denoted by θ_i and the within-source covariance matrix by U and $(\mathbf{X}_{ij} \mid \theta_i, U) \sim N(\theta_i, U), i = 1, \ldots, m, j = 1, \ldots, n.$

For between-source variation, the mean vector between sources i is denoted by μ and the between-source covariance matrix by C and $(\theta_i \mid \mu, C) \sim N(\mu, C), i = 1, \ldots, m..$

The means $(\mathbf{Y}_l \mid \theta_l, D_l) \sim N(\theta_l, D_l)$ where $D_l = n_l^{-1} U$ and for between-source normality, $(\mathbf{Y}_l \mid \mu, C, D_l) \sim N(\mu, C + D_l),\ l = 1, 2.$

The value of the evidence is the ratio of

$$\mid 2\pi\{(n_1 + n_2)U^{-1} + C^{-1}\}^{-1} \mid^{1/2} \exp\{-\frac{1}{2}(H_2 + H_3)\}$$

to

$$\mid 2\pi C \mid^{-1/2} \mid 2\pi\{n_1 U^{-1} + C^{-1}\}^{-1} \mid^{1/2} \mid 2\pi\{n_2 U^{-1} + C^{-1}\}^{-1} \mid^{1/2} \times \exp\{-\frac{1}{2}(H_4 + H_5)\} \quad (19)$$

where

$$
\begin{aligned}
H_2 &= (\mathbf{y}^* - \mu)^T \left(\frac{U}{(n_1 + n_2)} + C \right)^{-1} (\mathbf{y}^* - \mu), \\
H_3 &= (\bar{\mathbf{y}}_1 - \bar{\mathbf{y}}_2)^T (D_1 + D_2)^{-1} (\bar{\mathbf{y}}_1 - \bar{\mathbf{y}}_2), \\
H_4 &= (\mu - \mu^*)^T \{(D_l + C)^{-1} + (D_2 + C)^{-1}\} (\mu - \mu^*), \\
H_5 &= (\bar{\mathbf{y}}_1 - \bar{\mathbf{y}}_2)^T (D_1 + D_2 + 2C)^{-1} (\bar{\mathbf{y}}_1 - \bar{\mathbf{y}}_2), \\
\mathbf{y}^* &= \frac{n_1 \bar{\mathbf{y}}_1 + n_2 \bar{\mathbf{y}}_2}{n_1 + n_2}, \\
\mu^* &= \{(D_1 + C)^{-1} + (D_2 + C)^{-1}\}^{-1} \{(D_1 + C)^{-1} \bar{\mathbf{y}}_1 + (D_2 + C)^{-1} \bar{\mathbf{y}}_2\}.
\end{aligned}
$$

The notation is chosen to match that in Aitken and Lucy [2004][1]

The form of presentation is also chosen to be comparable with the univariate case described in Lindley [1977]. This emphasises the factors for rarity and similarity. The terms H_2 and H_4 are measures of rarity of means of the control and recovered measurements, first weighted by sample sizes and second weighted by covariances. The terms H_3 and H_5 are measures of similarity of the control and recovered measurements.

- Likelihood ratio with the assumption of constant within-source variation and kernel density estimation of between-source variation ; the formula for the likelihood ratio is not given here, for reasons of space, but is available in Aitken and Lucy [2004].
- Likelihood ratio when the assumption of the constant within-source variability is relaxed; see (Bozza et al. [2008]).

Consider background data of p-variables, with m groups and n_i measurements $\{\mathbf{z_{ij}} = (\mathbf{x_{ij1}}, \ldots, \mathbf{x_{ijp}}, \mathbf{i} = \mathbf{1}, \ldots, \mathbf{m};\ \mathbf{j} = \mathbf{1}, \ldots, \mathbf{n_i})\}$ in each group. Denote the mean vector within-group i by θ_i and the matrix of within-group variances and covariances by W_i and let $\psi = (\theta, W)$ with $\theta = (\theta_1, \theta_2)$ and $W = (W_1, W_2)$. Given θ_i and W_i, the distribution of Z_{ij} is taken to be Normal with $Z_{ij} \sim N(\theta_i, W_i)$. The distribution of the within-group mean θ is taken to be Normal, such that $\theta_i \sim N(\mu, B),\ i = 1.\ldots, m$. The distribution of the within-group matrix W is taken to be an inverted Wishart distribution, such that $W_i \sim IW(U, n_w),\ i = 1, \ldots, m$ where the number of degrees of freedom n_w is chosen to reduce the variability of the Wishart distribution.

A number n of measurements are available: n_1 measurements $\mathbf{y_1} = (y_{11}, \ldots, y_{1n_1})$ from a recovered source and n_2 measurements $\mathbf{y_2} = (y_{21}, \ldots, x_{2n_2})$ from a control source; $n_1 + n_2 = n$ and let $\mathbf{y}$ denote $(\mathbf{y}_1, \mathbf{y_2})$ [2].

Consider the proposition that the control and recovered measurements have the same source. Then $\theta_1 = \theta_2$ and $W_1 = W_2$. The density function of the data is

$$
f(\mathbf{y} \mid \psi, H_1) = \prod_{l=1}^{2} \prod_{j=1}^{n_l} (2\pi)^{-p/2} \mid W \mid^{1/2} \exp\Big\{ -\frac{1}{2} (\mathbf{y}_{lj} - \theta)' W^{-1} (\mathbf{y}_{lj} - \theta) \Big\}.
$$

The prior density of ψ is

[1] The notation of $\mathbf{x}$ for training data and $\mathbf{y}_1$ and $\mathbf{y}_2$ for control and recovered data is used here for consistency with Aitken and Lucy [2004] in contrast to $\mathbf{z}, \mathbf{x}$ and $\mathbf{y}$ in the rest of the paper. Also, H_1 denotes $\sum_{l=1}^{2} trace(S_l U^{-1})$ where $S_l = \sum_{j=1}^{n_l} (\mathbf{y}_{lj} - \bar{\mathbf{y}}_l)(\mathbf{y}_{lj} - \bar{\mathbf{y}}_l)^T$, an expression used in intermediate calculations but not in the final result.

[2] For notational convenience, both control and recovered data are denoted with $\mathbf{y}$; often $\mathbf{x}$ denotes control data and $\mathbf{y}$ denotes recovered data.

$$\pi(\psi \mid H_1) = (2\pi)^{-p/2} \mid B \mid^{-1/2} \exp\left\{ -\frac{1}{2}(\theta-\mu)' B^{-1}(\theta-\mu)\right\} \times \frac{c \mid U \mid^{(n_w-p-1)/2}}{\mid W \mid^{n_w/2}} \exp\left\{ -\frac{1}{2} tr(W^{-1}U)\right\}.$$

90 The complete conditional density of θ is then

$$\pi(\theta \mid W, \mathbf{y}) \propto \exp\left[-\frac{1}{2}\left\{ \sum_{l=1}^{2}\sum_{j=1}^{n_l}(\mathbf{y}_{lj}-\theta)' W^{-1}(\mathbf{y}_{lj}-\theta) + (\theta-\mu)' B^{-1}(\theta-\mu)\right\}\right]. \quad (20)$$

91 The complete conditional density of W is

$$\pi(W \mid \theta, \mathbf{y}) \propto \mid W \mid^{-n/2} \exp\left\{ -\frac{1}{2}\sum_{l=1}^{2}\sum_{j=1} n_l(\mathbf{y}_{lj}-\theta)' W^{-1}(\mathbf{y}_{lj}-\theta)\right\} \times \mid W \mid^{-n_w/2} \exp\left\{ -\frac{1}{2} tr(W^{-1}U)\right\}. \quad (21)$$

92 The function $\pi(\psi \mid \mathbf{y}, H_k)$ is obtained from (20) and (21) with the use of Gibbs sampling.
Consider the proposition H_2 that the control and recovered measurements have different sources. The density function of the data $\mathbf{y}$ is then

$$f(\mathbf{y} \mid \psi, H_2) = \prod_{l=1}^{2}\left[\prod_{j=1}^{n_l}(2\pi)^{-p/2} \mid W_l \mid^{-1/2} \exp\left\{ -\frac{1}{2}(\mathbf{y}_{lj}-\theta_l)' W_l^{-1}(\mathbf{y}_{lj}-\theta_l)\right\}\right]$$

93 The complete conditional densities of θ and W are, for $l = 1, 2$,

$$\pi(\theta_l \mid W_l, \mathbf{y}) \propto \exp\left\{ -\frac{1}{2}(\theta_l-\mu_l^*)' B_l^{*-1}(\theta_l-\mu_l^*)\right\}. \quad (22)$$

94 and

$$\pi(W_l \mid \theta_l, \mathbf{y}) \propto \mid W_l \mid^{-(n_l+n_w)/2} \exp\left\{ -\frac{1}{2} \exp\left(-\frac{1}{2} tr\left[W_l^{-1}\{n_l(\theta_l-\bar{\mathbf{y}}_l)(\theta_l-\bar{\mathbf{y}}_l)' + S_l U\}\right]\right)\right. . \quad (23)$$

95 with

$$\begin{aligned} B_l^* &= (B^{-1} + n_l W_l^{-1})^{-1}, \\ \mu_l^* &= B_l^*(B^{-1}\mu + n_l W_l^{-1}\bar{\mathbf{y}}_l), \\ S_l &= \sum_{j=1}^{n_l} (\bar{y}_{lj} - \bar{\mathbf{y}}_l)'(\bar{y}_{lj} - \bar{\mathbf{y}}_l), \end{aligned}$$

where $\bar{\mathbf{y}}_l = \sum_{j=1}^{n_l} \mathbf{y}_{lj}/n_l$.

The function $\pi(\psi \mid \mathbf{y}, H_2)$ is obtained from (22) and (23) with the use of Gibbs sampling.

The marginal likelihood is then given from the equation

$$m(\mathbf{y} \mid H_k) = \frac{f(\mathbf{y} \mid \psi, H_k)\pi(\psi \mid H_k)}{\pi(\psi \mid \mathbf{y}, H_k)}. \quad (24)$$

Further details are available in Bozza et al. [2008]

REFERENCES

J. Aitchison and I. Dunsmore. *Statistical Prediction Analysis*. Cambridge University Press, Cambridge, 1975.

J. Aitchison, J.D.F. Habbema, and J.W. Kay. A critical comparison of two methods of statistical discrimination. *Applied Statistics, Journal of the Royal Statistical Society, Series C*, 26:15–25, 1977.

C. G. G. Aitken and D. Lucy. Evaluation of trace evidence in the form of multivariate data. *Journal of the Royal Statistical Society. Series C (Applied Statistics)*, 53:109–122, 2004.

C.G.G. Aitken and F. Taroni. *Statistics and the Evaluation of Evidence for Forensic Scientists*. Wiley, Chichester, 2 edition, 2004.

I. Alberink, A. Bolck, and S. Menges. Posterior likelihood ratios for evaluation of forensic trace evidence given a two-level model on the data. *Journal of Applied Statistics*, 40:2579–2600, 2013. doi: 10.1080/02664763.2013.822056.

S. Bozza, F. Taroni, R. Marquis, and M. Schmittbühl. Probabilistic evaluation of handwriting evidence: likelihood ratio for authorship. *Journal of the Royal Statistical Society: Series C (Applied Statistics)*, 57: 329–341, 2008. doi: 10.1111/j.1467-9876.2007.00616.x.

I.W Evett, P.E. Cage, and C.G.G. Aitken. Evaluation of the likelihood ratio for fibre transfer evidence in criminal cases. *Applied Statistics*, 36:174–180, 1987.

D. V. Lindley. A problem in forensic science. *Biometrika*, 64(2):207–213, 1977. doi: 10.1093/biomet/64.2.207. URL `http://biomet.oxfordjournals.org/content/64/2/207.abstract`.

J.C. Wakefield, A.M. Skene, A.F.M. Smith, and I.W. Evett. The evaluation of fibre transfer evidence in forensic science: a case study in statistical modelling. *Applied Statistics*, 40:461–476, 1991.

G. Zadora, A. Martyna, D. Ramos, and C.G.G. Aitken. *Statistical analysis in forensic science: evidential value of multivariate physicochemical data*. John Wiley and Sons Ltd., Chichester, 2014.
